# Supplementary material for: From maps to models: A survey on the reliability of small studies of task-based fMRI
Source: Imaging Neurosci (Camb). 2026 Jan 7;4:IMAG.a.1076. doi: 10.1162/IMAG.a.1076 (PMC12779752; doi:10.1162/IMAG.a.1076)
Supplement: Supplementary Material [file IMAG.a.1076_supp.pdf]

5 Supplementary Material

5.1 Excluded Participants

The following 93 participants were excluded from analyses due to either QC issues or the presence of a twin in the included sample: 110613, 113417, 113821, 120010, 121719, 130518, 139637, 143830, 146836, 168139, 175035, 176239, 185038, 189652, 199958, 201515, 202820, 385046, 401422, 415837, 433839, 462139, 465852, 469961, 644246, 656657, 688569, 723141, 767464, 872764, 943862, 965367, 969476, 987983, 994273, 433839, 103010, 113417, 116423, 120010, 121719, 127226, 130114, 143830, 169040, 185038, 189652, 202820, 204218, 329844, 385046, 401422, 462139, 469961, 644246, 688569, 723141, 908860, 943862, 969476, 971160, 196952, 748662, 809252, 144428, 186545, 192237, 223929, 320826, 644044, 822244, 870861, 947668, 102614, 111009, 111514, 115017, 121416, 130821, 138332, 179952, 299760, 300618, 392750, 406432, 429040, 633847, 662551, 679770, 688569, 693461, 815247, 142626

5.2 Tasks

5.2.1 HCP

The following task descriptions are paraphrased from the details provided by Barch et al. (2013).

Working Memory In the working memory runs, participants (494) completed N-back tasks (Drobyshevsky et al., 2006). Each of the two runs contains eight stimulus blocks consisting of ten trials ($2\,\mathrm{s}$ stimulus presentation, $500\,\mathrm{ms}$ ITI) and four fixation blocks ($15\,\mathrm{s}$ each). Within each run, four different stimulus types (faces, places, tools, and body parts) were presented in separate blocks. Task type was indicated at the start of each block with a $2.5\,\mathrm{s}$ cue. Each block contains two targets and 2–3 non-target lures. Half of the blocks for each stimulus type used a 2-back task, and the other half a 0-back task. Total scan time was 5:01 (405 frames).

The design matrix included eight task-related predictors, one for each stimulus type in each of the N-back conditions. Each predictor covered the period from the onset of the cue to the offset of the final trial. Our analyses considered a contrast selecting for the 2-back parameter, the 0-back parameter, and a comparison of the 2-back minus 0-back parameters.

Motor In each of two runs, participants (492) were asked to move different body parts (Buckner et al., 2011; Thomas Yeo et al., 2011). The body part to move was indicated by visual cues presented at the start of each block (one $3\,\mathrm{s}$ cue for each $12\,\mathrm{s}$ block). Cues indicated that participants should either tap their left or right fingers, squeeze their left or right toes, or move their tongue (only one type of motion was asked for in each block). Each run contained two blocks of tongue movements, two blocks of each hand movement, two blocks of each foot movement, and three additional blocks of fixation (each $15\,\mathrm{s}$). Total scan time was 3:34 (284 frames).

The design matrix included five task-related predictors, each covering the duration of the 10 movement trials ($12\,\mathrm{s}$). The cue was modeled separately. Our analyses included a contrast selecting for the cue, one selecting for the average motion, and a comparison of the average motion minus the baseline.

Gambling In this task, participants (494) were asked to guess the number on a hidden card to win or lose money (Delgado et al., 2000). They were informed that the number ranged from 1 to 9, and that they should guess whether the hidden number was greater or less than 5. Guesses were realized by pressing one of two buttons. The task was presented in blocks of eight trials that comprised either mostly rewards (6 reward trials interleaved with either one neutral and one loss trial, two neutral trials, or two loss trials) or mostly losses (6 loss trials interleaved with either one neutral and one reward trial, two neutral trials, or two reward trials). After responding, participants were given feedback. On reward trials, the feedback was a green up arrow with $1, on loss trials, it was a red down arrow with $0.50, and on neutral trials, it was the number 5 with a gray, double-headed arrow. In each of the two runs, there were two mostly reward and two mostly loss blocks, interleaved with four fixation blocks (each $15\,\mathrm{s}$). Total scan time was 3:12 (253 frames).

The design matrix included two task-related predictors that modeled the mostly reward and mostly punishment blocks, each covering the duration of 8 trials ($28\,\mathrm{s}$). Our analyses relied on contrasts for the reward parameter, the punish parameter, and the reward minus punish parameters.

Language In each run of this task, participants (483) listened to eight blocks of stimuli, four of which consisted of short stories and four of which consisted of arithmetic (Binder et al., 2011). Blocks averaged $40\,\mathrm{s}$ and the two stimulus types were interleaved. After each block, participants were presented with a two-alternative forced-choice question that either asked about the story or the result of the arithmetic. Total scan time was 3:57 (316 frames).

The design matrix included two predictors that corresponded to the two types of stimuli. Our analyses relied on contrasts for the story parameter, the math parameter, and the difference between math and

story.

Relational In this task, participants (481) were presented with sets of stimuli and asked to discern whether they matched or differed according to pre-specified rules (Smith et al., 2007). Stimuli varied by shape and texture. In a relational condition, two pairs of stimuli were presented. One pair serves as a reference, and the stimuli in that pair differ along one of the two dimensions. Participants first identified the mismatching dimension and then determined whether the other pair of stimuli differed along the same dimension. In a matching condition, a single pair of object stimuli was presented along with a third object and a word. The word identified one of the two features, and participants had to determine whether the identified feature in the third stimulus matched the feature value for either of the paired stimuli. Relational stimuli were presented for $3500\,\mathrm{ms}$ (ITI: $500\,\mathrm{ms}$) and matching stimuli for $2800\,\mathrm{ms}$ (ITI: $400\,\mathrm{ms}$). Stimuli were presented in three blocks that each contained five trials, and the stimulus blocks were interspersed with three fixation blocks ($16\,\mathrm{s}$). Total scan time was 3:57 (316 frames).

The design matrix for this task included two predictors, one for each of the two conditions ("match" and "relation"). Our analyses relied on contrasts for the matching minus relation parameters.

Social The social task used stimuli derived from Castelli et al. (2013) and Wheatley et al. (2007). The stimuli were videos of shapes that moved either randomly or with a set of specified interactions ($20\,\mathrm{s}$ per video). After each video, participants (486) selected one of three responses indicating whether they observed the objects interacting. There were five blocks of trials per run (conditions balanced across the two runs). Total scan time was 3:27 (274 frames).

The design matrix for this task contained two predictors, one for each of the "theory of mind" and "random" conditions. Our analyses used contrasts that selected the subtraction of the theory of mind from the random parameter.

Emotion In this task, participants (374) were presented with blocks of trials that either asked them to decide which of two faces shown at the bottom of the screen matched the face at the top, or which of two shapes presented at the bottom matched the shape at the top. The faces had either angry or fearful expressions. Trials are presented in blocks of 6 trials ($2\,\mathrm{s}$ stimulus presentation, $1\,\mathrm{s}$ ITI) of the same task ("face" or "shape"). Each block was preceded by a $3\,\mathrm{s}$ task cue ("shape" or "face"). Each of the two runs includes three face blocks and three shape blocks. Total scan time was 2:16 (176 frames).

Two different task-related predictors were included in the design matrix, one corresponding to emotional

faces and the other to the shape control condition. Each predictor covered a $21\,\mathrm{s}$ duration composed of a cue and six trials. A linear COPE comparing emotional faces vs shapes was used for further analysis.

5.2.2 UKB

The following task descriptions are paraphrased from the details provided by Alfaro-Almagro et al. (2018).

Emotion The single UKB task is very similar to the Emotion task in the HCP. Both block types (faces and shapes) were each presented for five, $21\,\mathrm{s}$ blocks. Total scan time was 4:13 (332 frames). Analyses focused on the Faces-Shapes contrast.

5.3 Impact of Population Size on Simulations

As noted in the main text, the absolute values reported for reliability may exhibit a bias. Many of the analyses relied on a subset of the HCP-YA dataset with fewer than 400 participants. From this population, studies were generated by repeatedly drawing individuals, and the main results consisted of summaries derived from the samples. Here, we outline the source of the bias.

The key issue relates to sample overlap. When generating studies that are close to the full population size, the participant composition of each sample will have a large overlap. For example, with a population of 100 and a sample of 90, bootstrap studies will share substantial participant overlap. By contrast, with a population of 1,000 and the same sample size (90), overlap is much smaller, and many participants will appear in relatively few studies. Greater overlap artificially inflates between-study similarity, biasing reliability estimates upward.

To assess the impact of this, a simulation study was conducted. We considered the effect of dataset size on the reliability of voxel-wise measurements of effect size. We generated 100 "full" datasets (each participant contributing 100 voxels, with effect sizes generated from a normal distribution with mean zero and standard deviation 0.1), each with M participants contributing $P = 100$ observations. For each full dataset, $B = 100$ bootstrap samples were generated, each containing $N = 16$ participants. Each bootstrap sample was then summarized (a voxelwise average across the N participants), and the summaries were compared to each other by calculating an intraclass correlation. The correlations, shown on the y-axis, were then summarized across each of the complete datasets (mean and two standard errors). As the population size increases, the estimated reliability decreases (Figure S1). This is consistent with the interpretation that overlap in small finite populations inflates reliability.

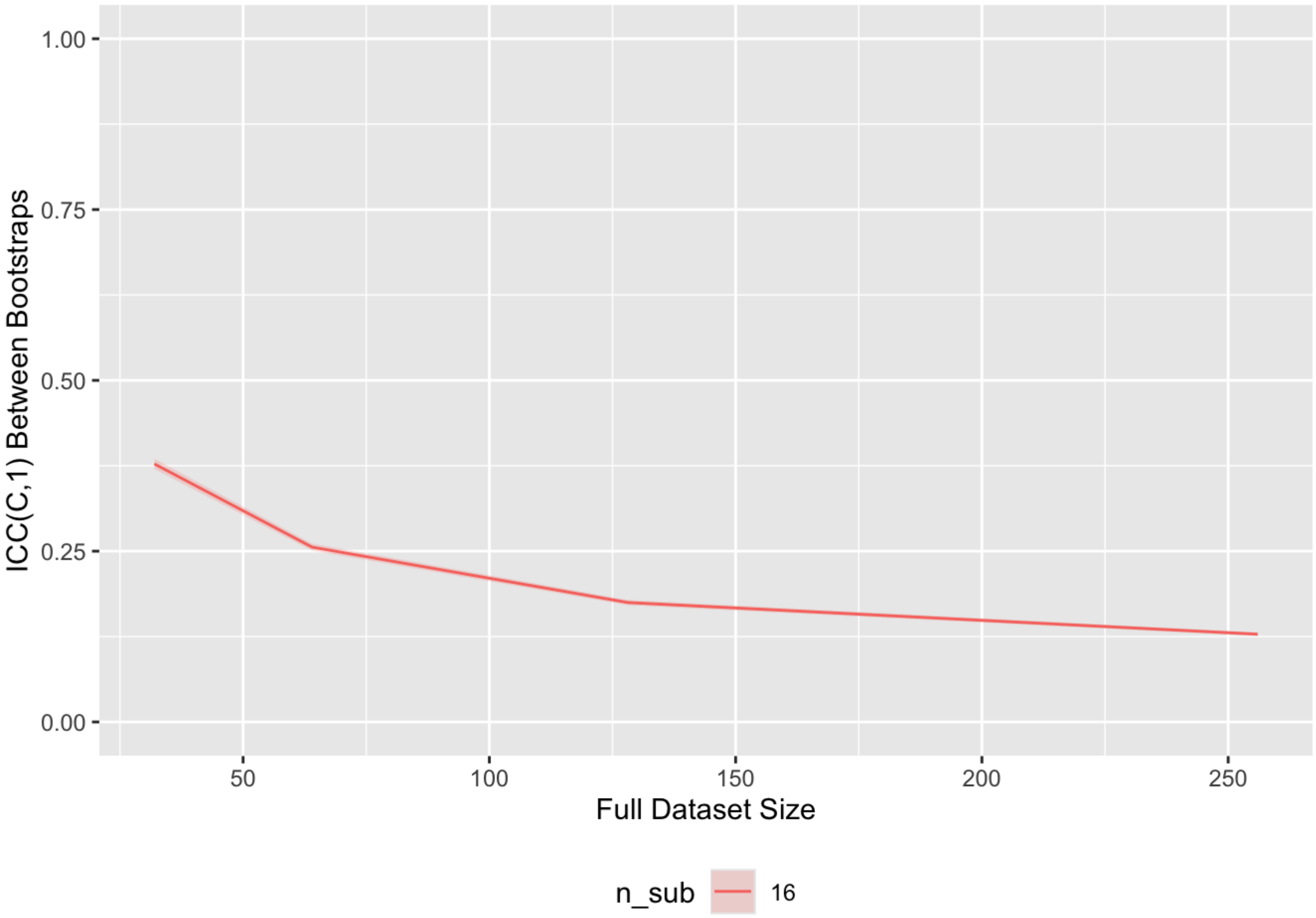

Figure S1: Estimated reliability of voxelwise effect sizes depends on sample size. The Intraclass Correlation Coefficient (ICC) was calculated as described in the main text.

5.4 Supplementary Figures

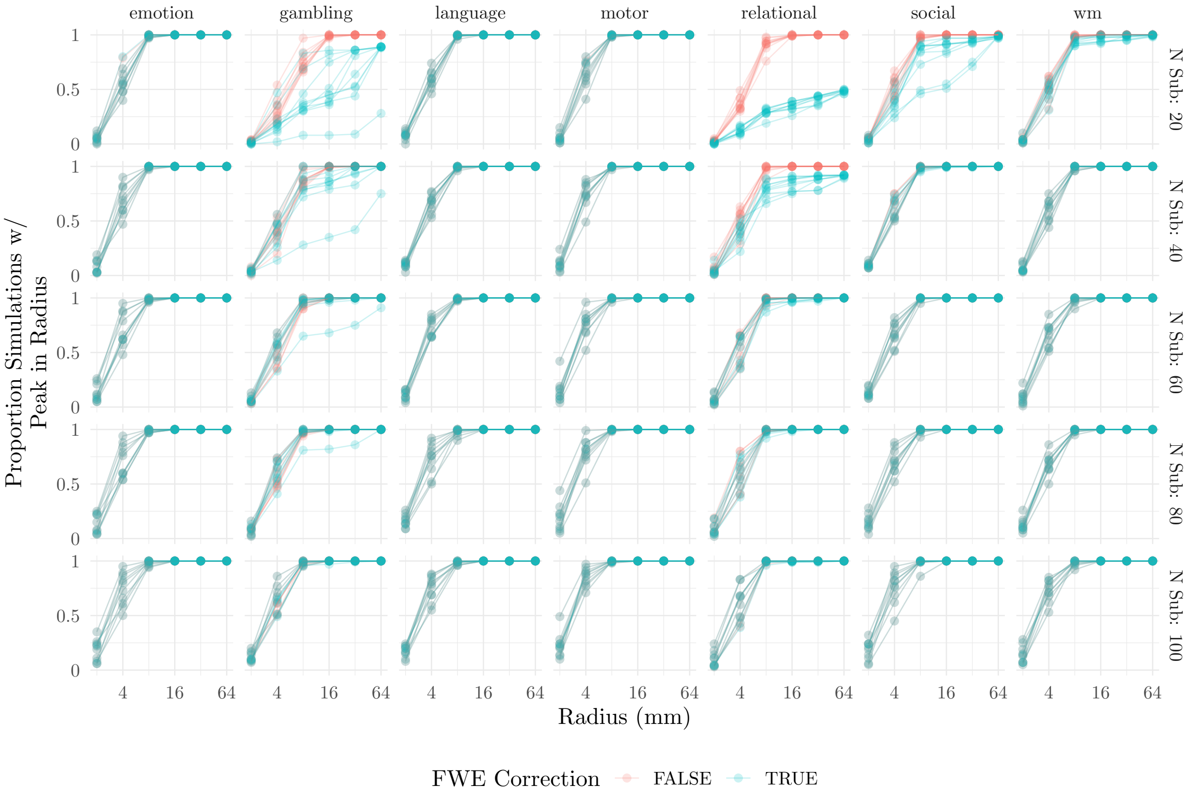

Figure S2: Volumetric peak localization with and without family-wise error rate correction using probabilistic threshold-free cluster enhancement (Spisák et al., 2019). Data plotted as in Figure 4. There is a clear effect of thresholding with family-wise error correction when sample sizes are 40 or 20, but at larger sample sizes, the effect is diminished. With more than 40 participants, there is only a minimal change in peak recovery for most of the largest peaks.

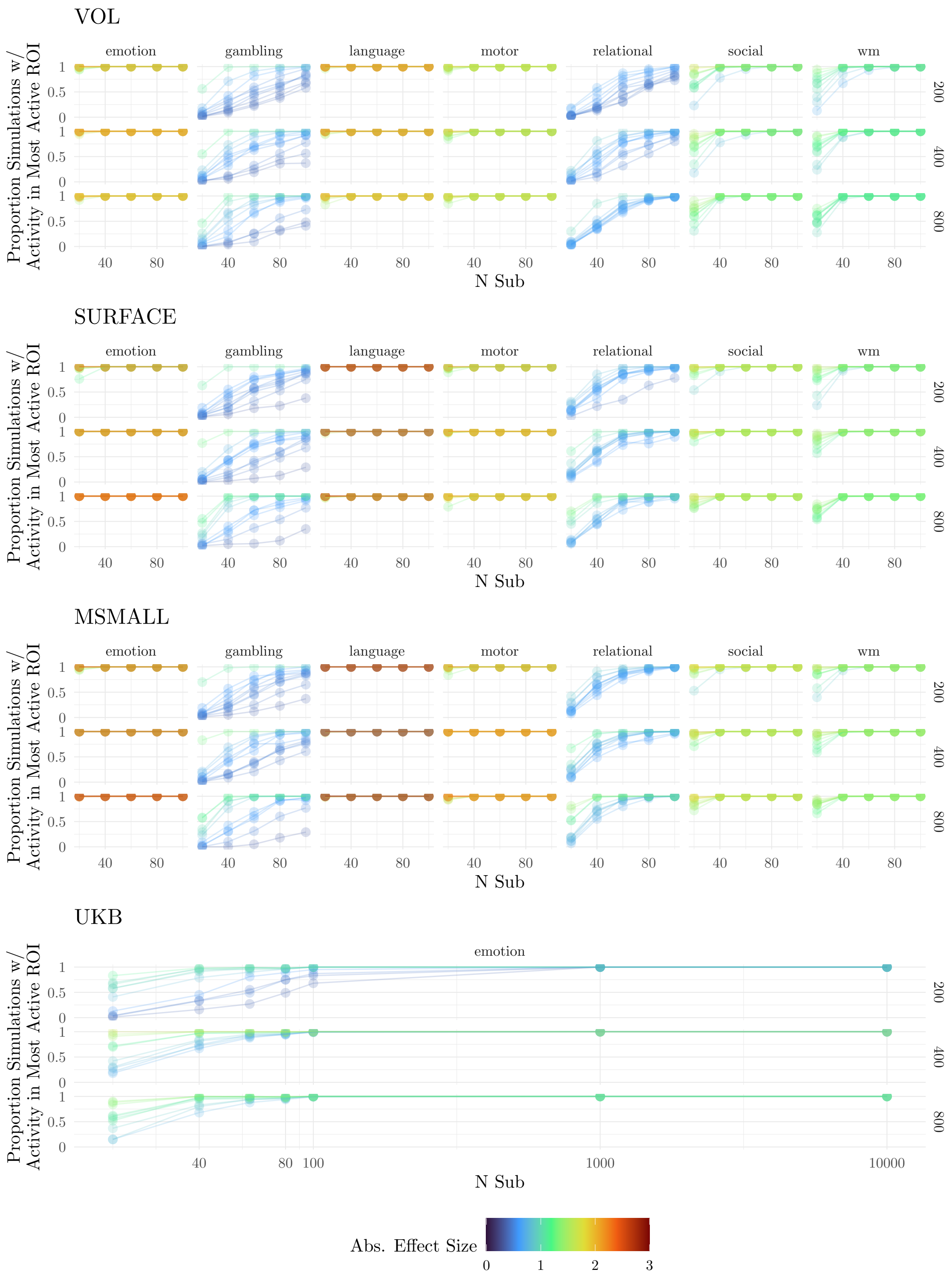

Figure S3: Recovery of gold standard across parcellations. Rows indicate the number of parcels within the atlas. Compare with Figure 2a.

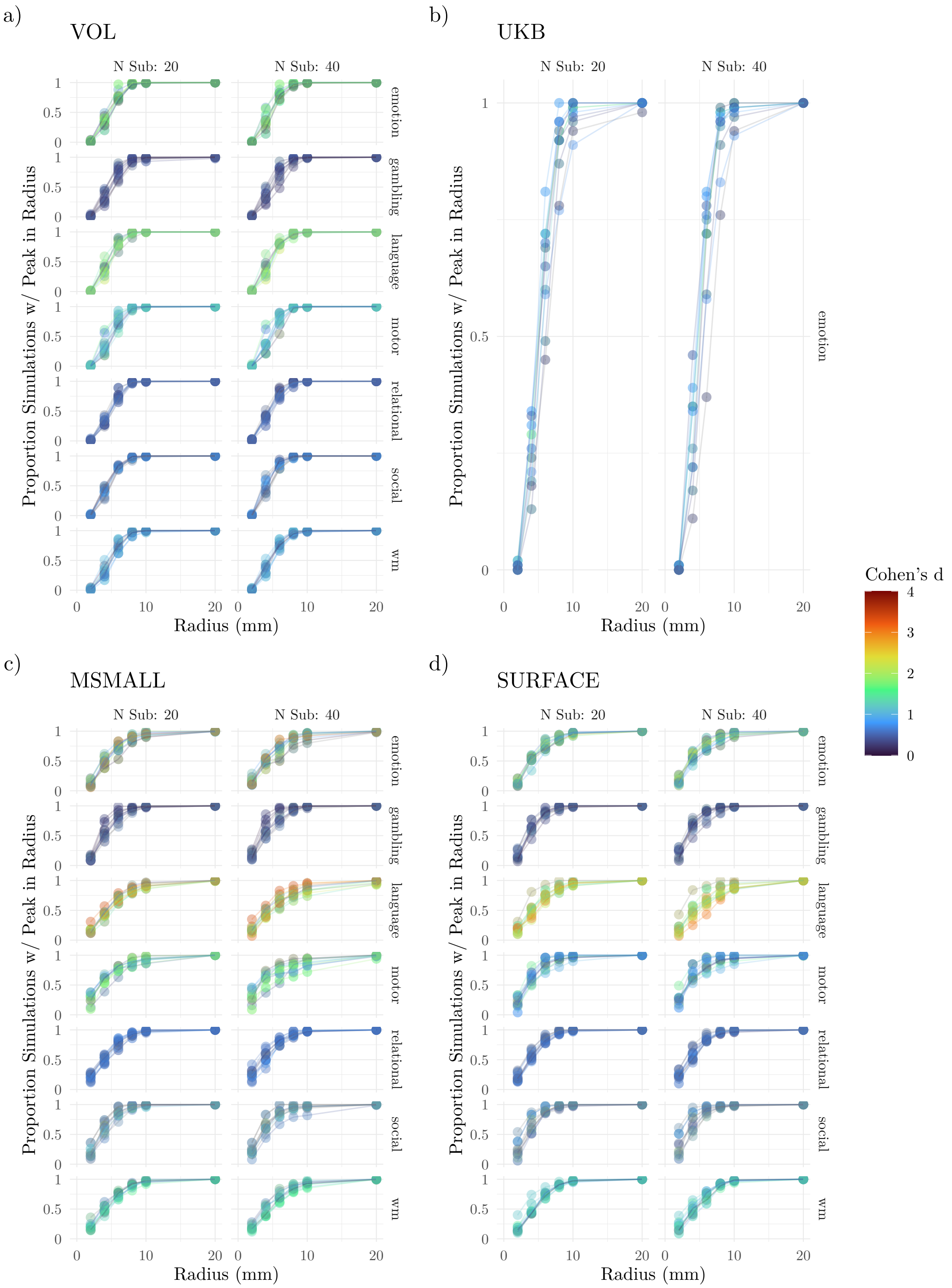

Figure S4: Figure plotted as in Figure 4, but peaks are defined within unthresholded maps.

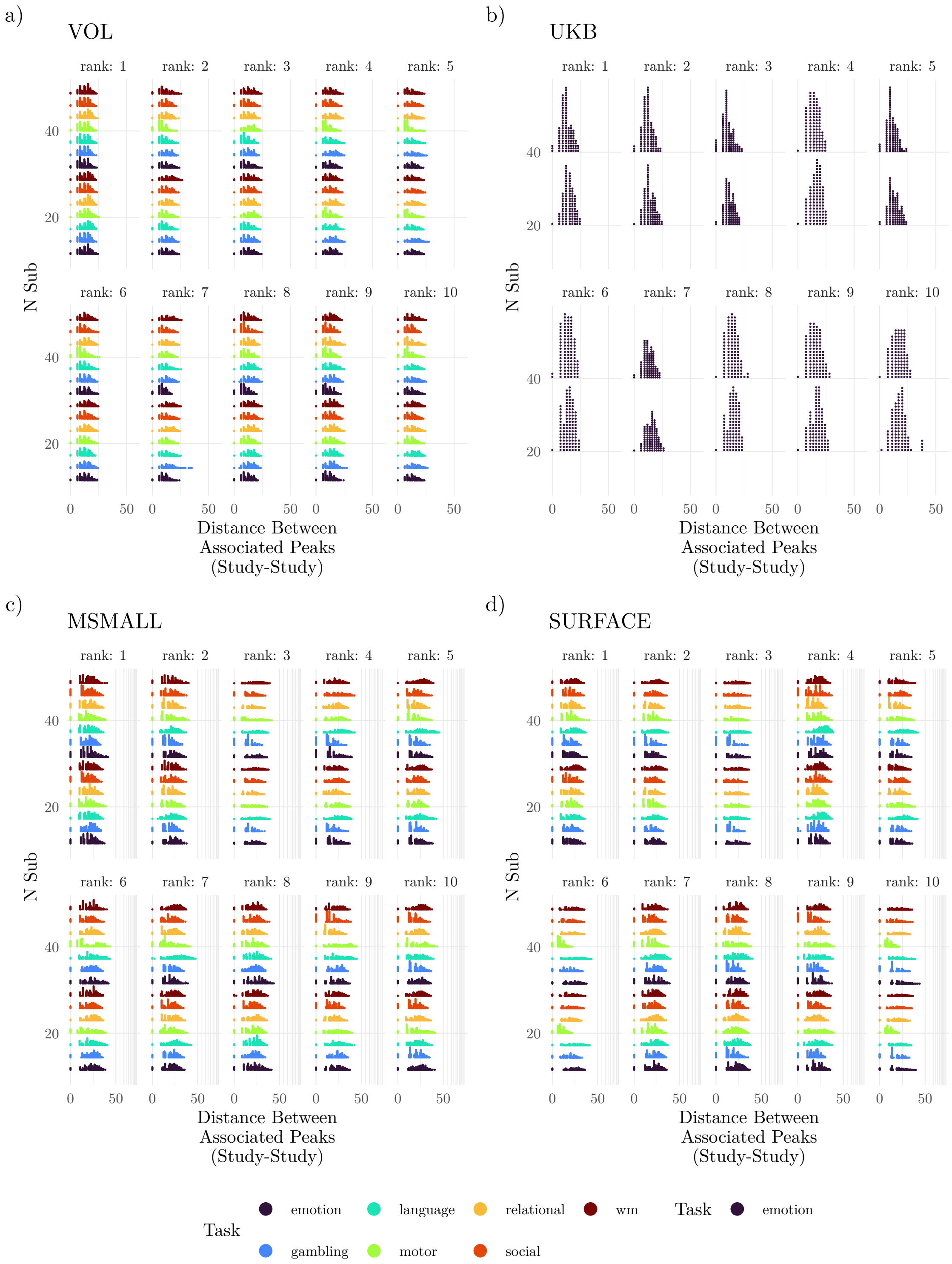

Figure S5: Figure plotted as in Figure 5, but peaks are defined within unthresholded maps.

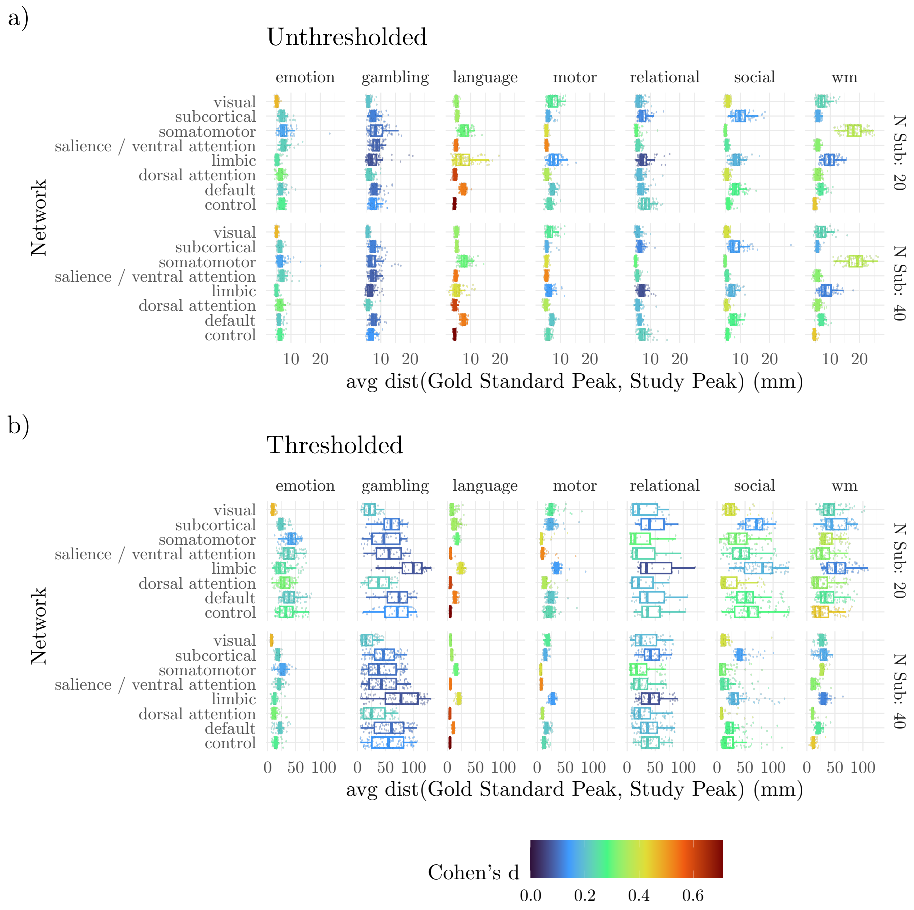

Figure S6: Distribution of average distances within Yeo7 networks. Studies are organized by sample size (rows) and task (columns). Peaks were selected from each gold standard map and labeled according to the Yeo7 networks (VOL only). Across all peaks within a network, the distance to the nearest study peak was calculated, and then these distances were averaged by study, network, task, and sample size. Points mark the distance to the nearest peak within each generated study, where the study maps were either unthresholded **(a)** or thresholded with family-wise error rate corrected $p < 0.05$ **(b)**. Colors indicate the average effect size of peak voxels in the gold standard for the given network.

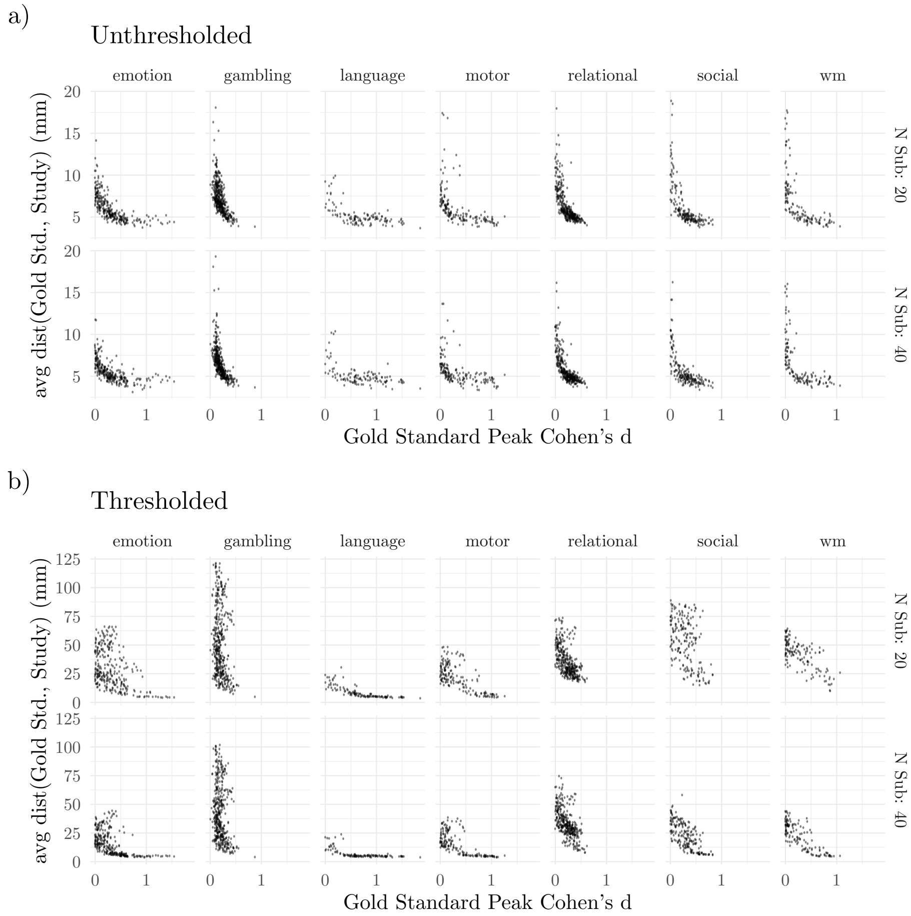

Figure S7: Average Distance from Gold Standard Peak by Effect Size. Individual points correspond to peaks in the gold standard. Averages were taken across studies and grouped by sample size. Note that only the VOL analyses are shown. Peaks are from maps that were either left unthresholded **(a)** or thresholded with family-wise error rate corrected $p < 0.05$ **(b)**.

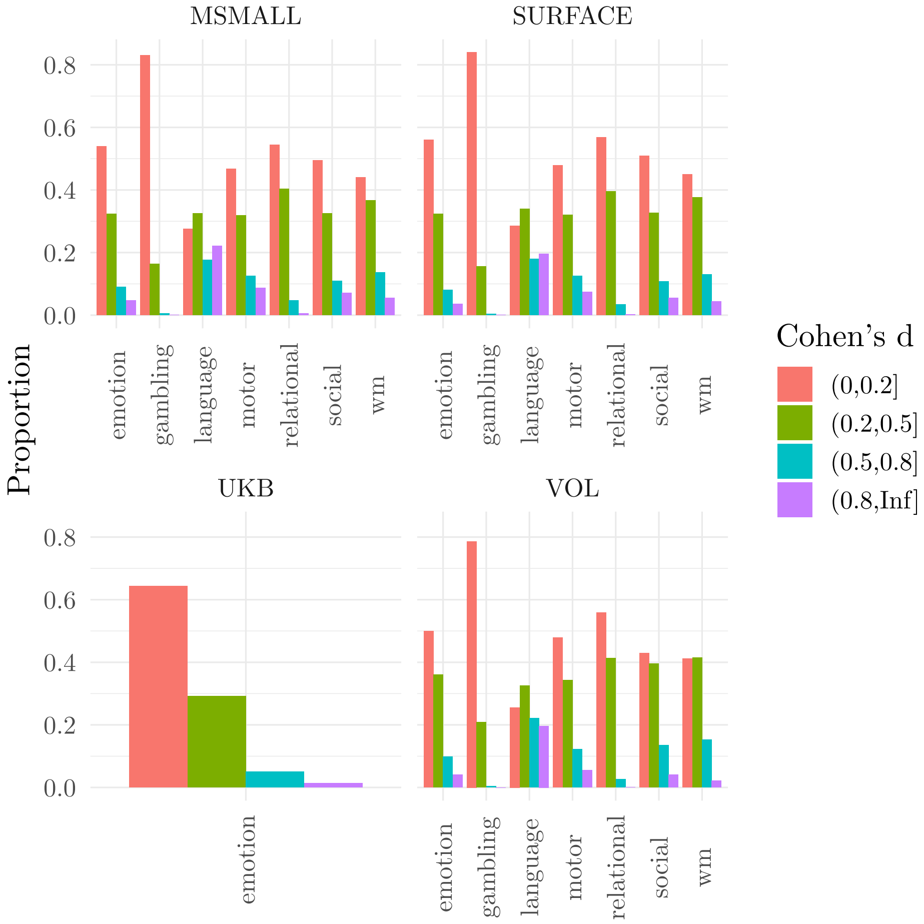

Figure S8: Voxel-wise effect sizes produced by each task. Categories were defined as specified in the Methods.

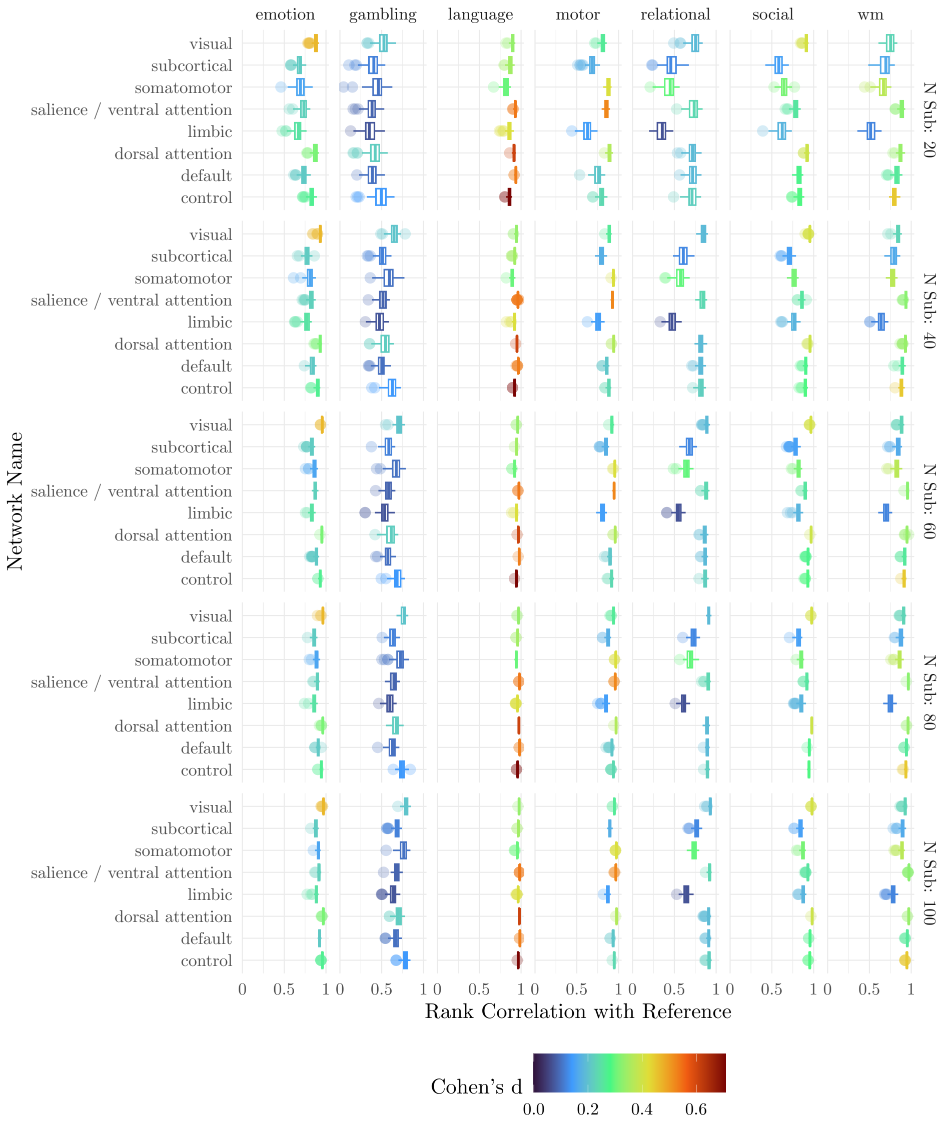

Figure S9: Recovery of gold standard by Yeo7 networks. Colors indicate the average effect size of voxels assigned to the network within the gold standard. Points correspond to sampled studies.

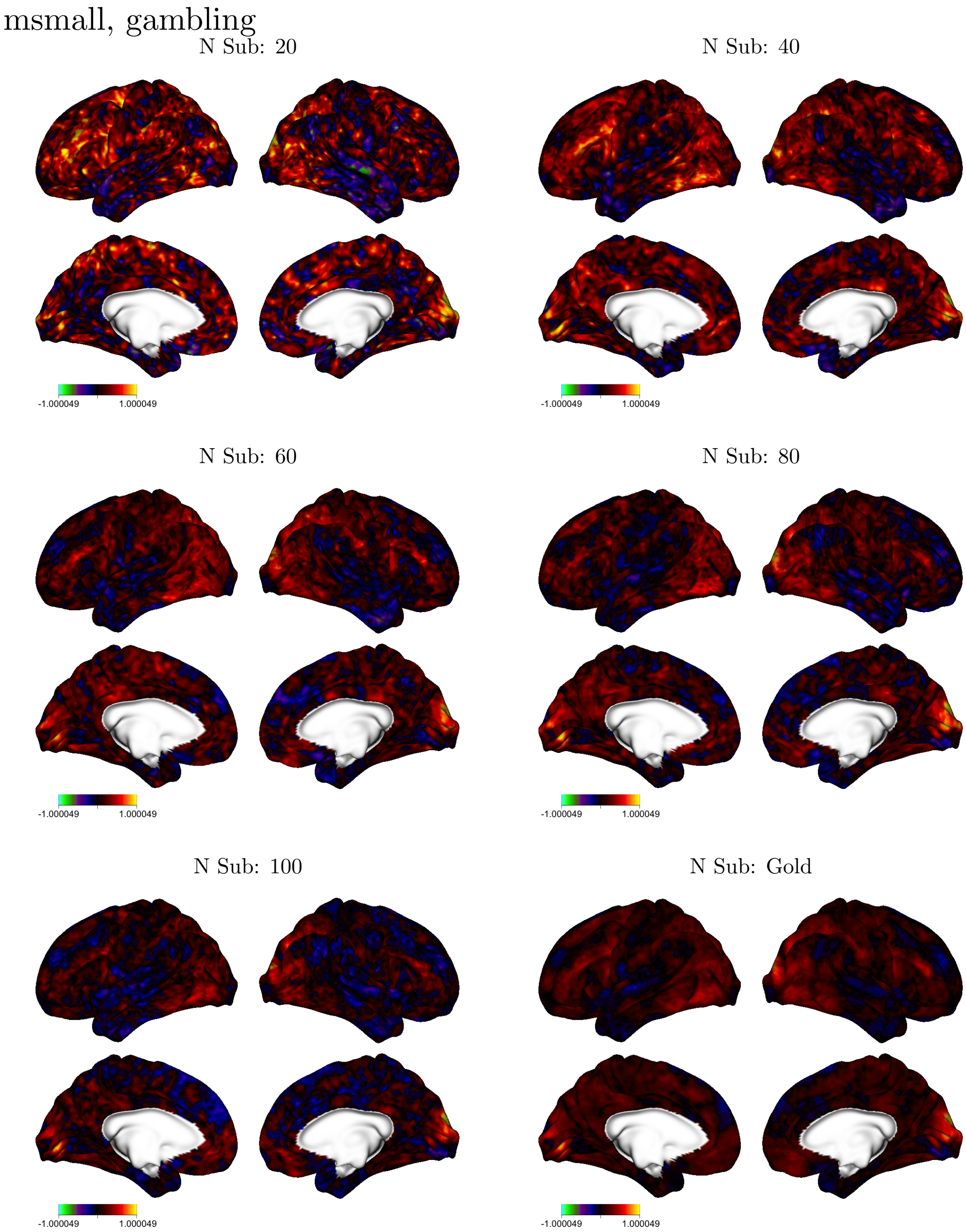

Figure S10: Effect Size in the Gambling Task. Data plotted as in Figure 6

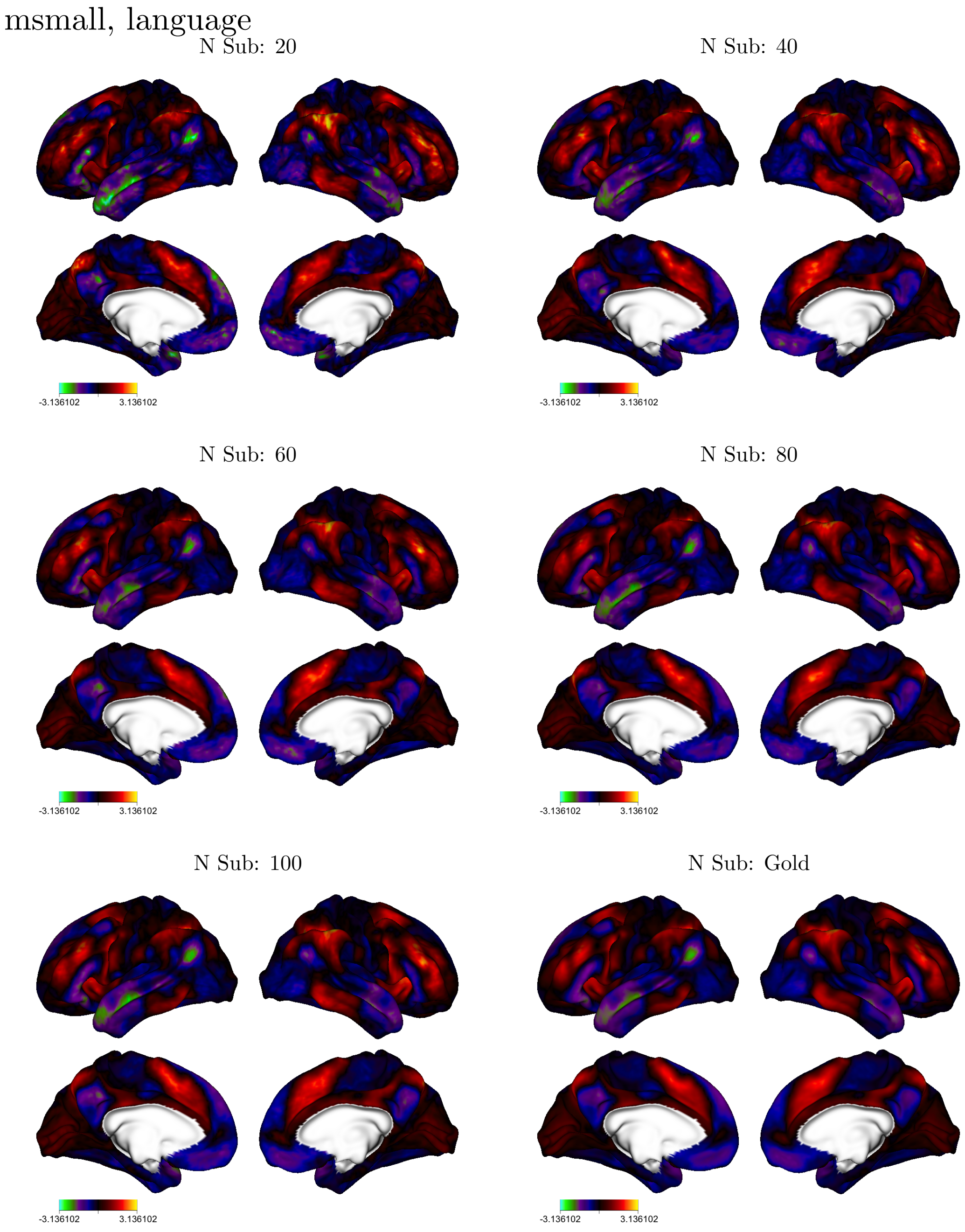

Figure S11: Effect Size in the Language Task. Data plotted as in Figure 6

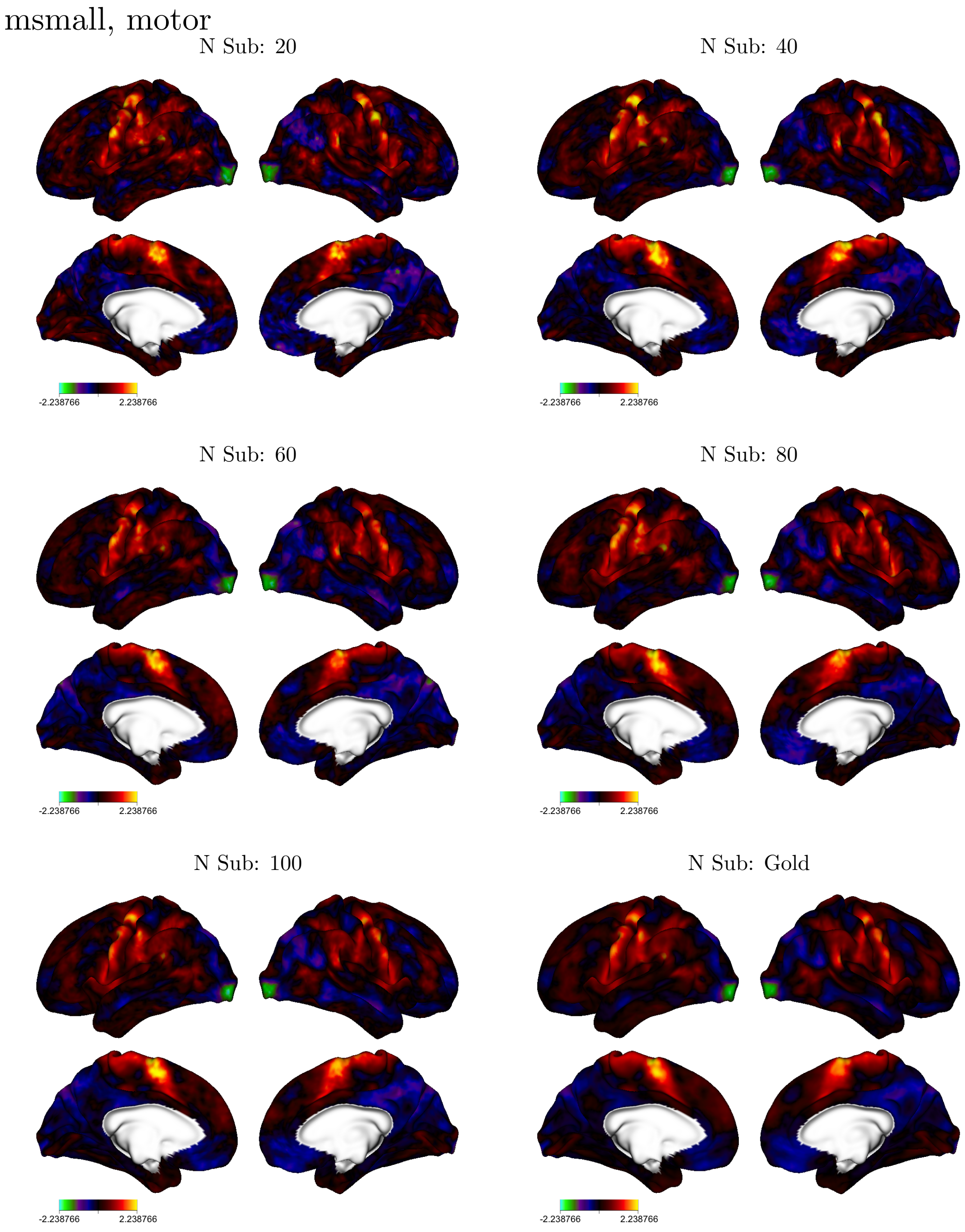

Figure S12: Effect Size in the Motor Task. Data plotted as in Figure 6

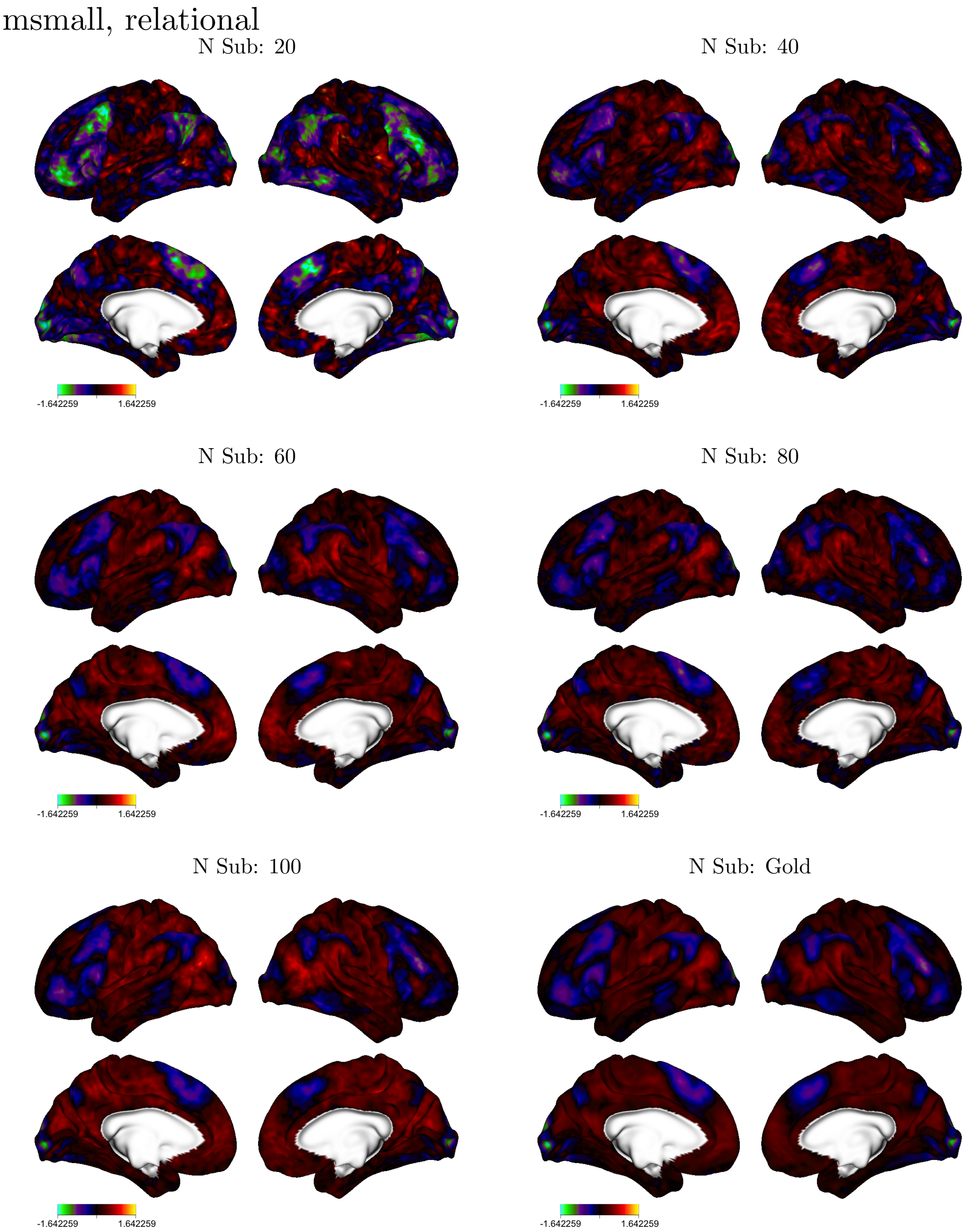

Figure S13: Effect Size in the Relational Task. Data plotted as in Figure 6

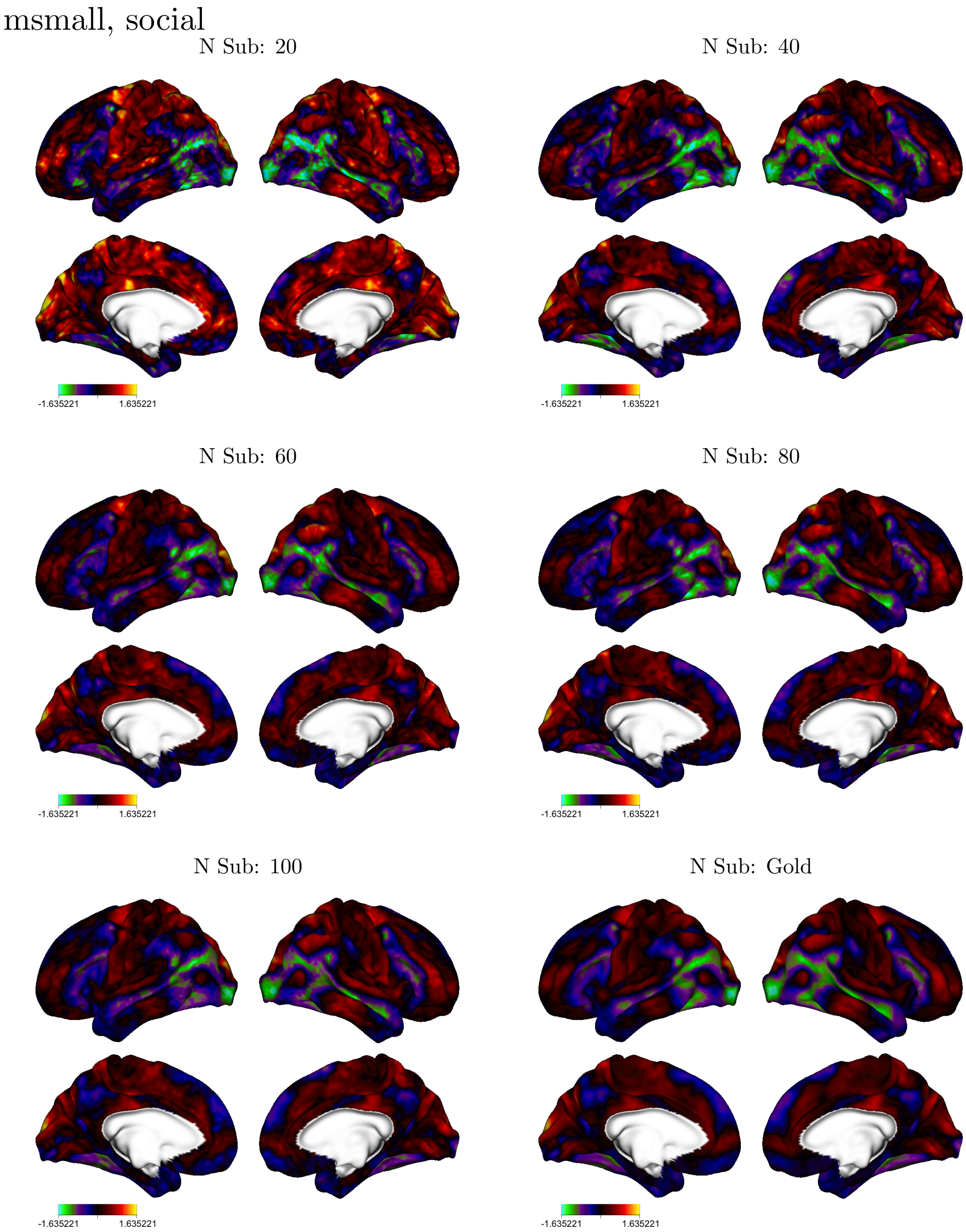

Figure S14: Effect Size in the Social Task. Data plotted as in Figure 6

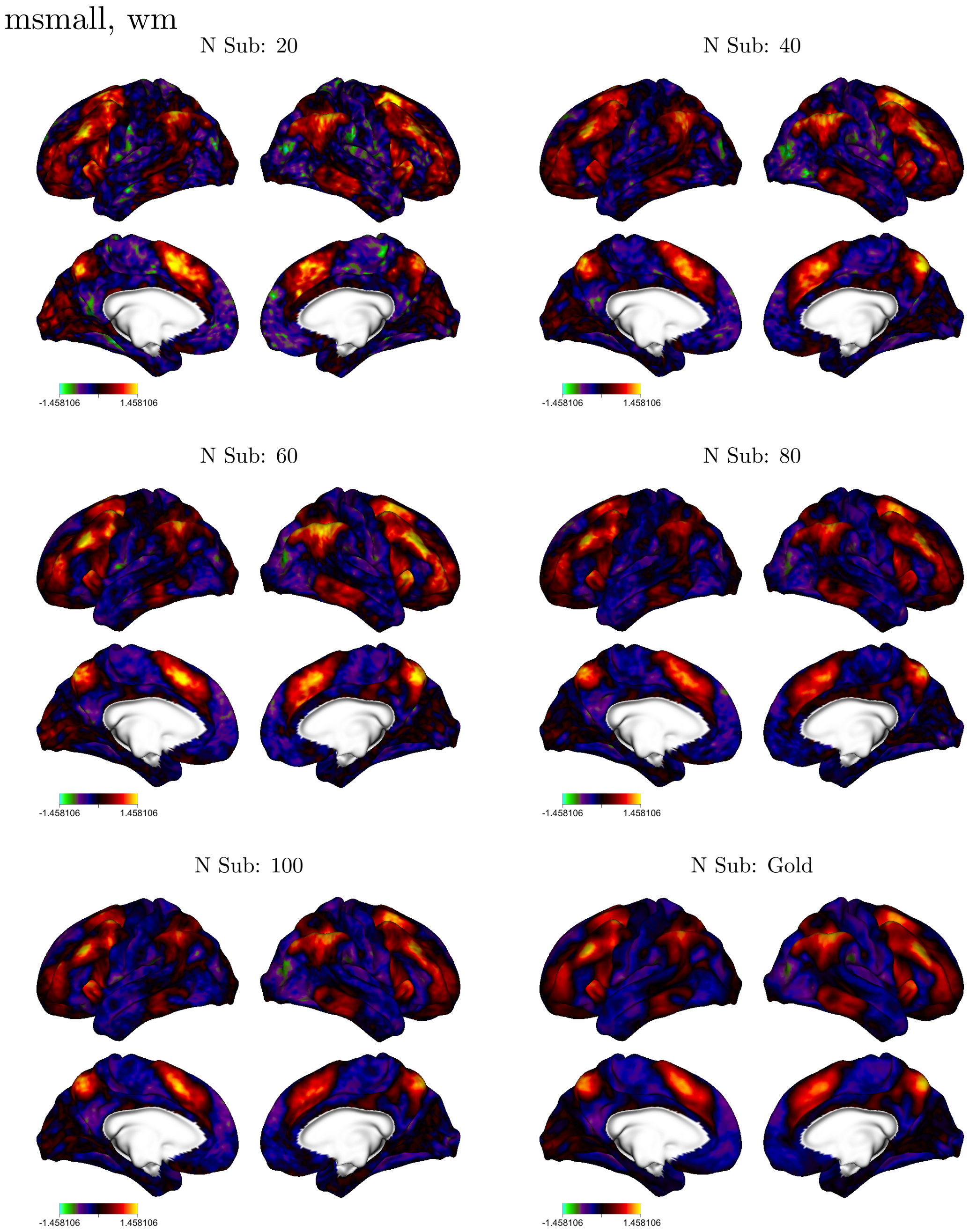

Figure S15: Effect Size in the Working Memory (WM) Task. Data plotted as in Figure 6

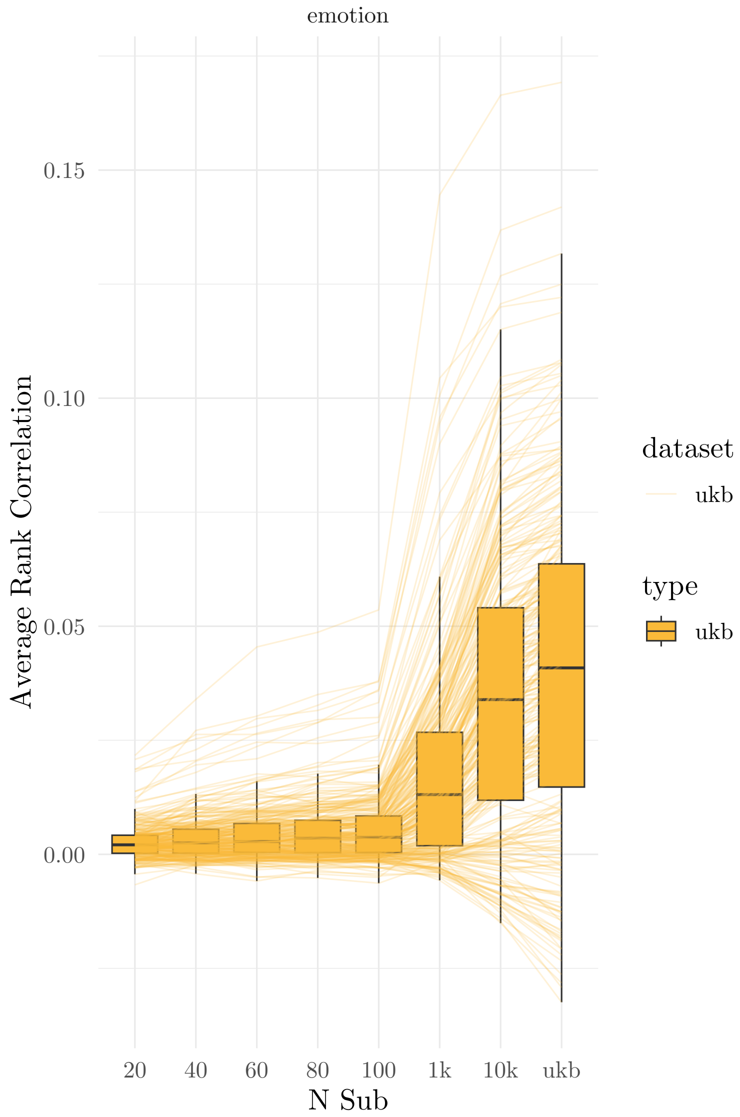

Figure S16: Model Performance with Alternative Features (Effect Size in 10 Highest ROI). Compare with Figure 8a.

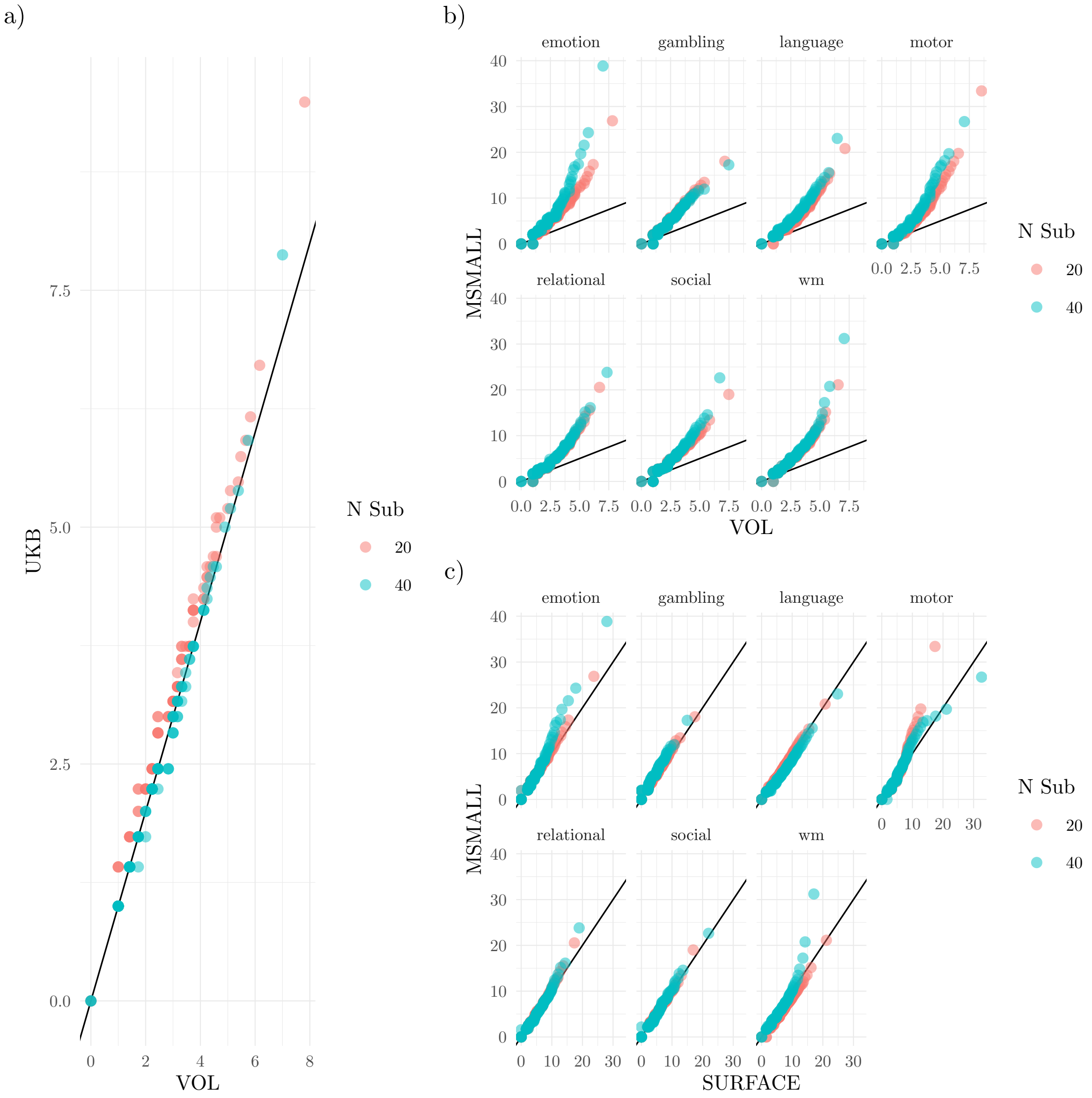

Figure S17: Differences in Peak Reliability Across Types. For each type, the distribution of distances for peaks associated with the largest peak activation (i.e., 4950 distances) was used to calculate an empirical cumulative density function. That function was then used to estimate the distribution percentiles, and these percentiles were plotted against one another. Solid lines mark the line of equality, so points above (below) the line indicate that the variable on the y-axis has greater (lower) reliability. a) VOL vs UKB. b) VOL vs MSMAll. c) Surface vs MSMAll.

Type	Task	N Sub	Proportion With Peak
MSMALL	gambling	20	0.45
MSMALL	gambling	40	0.84
MSMALL	relational	20	0.29
MSMALL	relational	40	0.67
MSMALL	social	20	0.29
MSMALL	social	40	0.96
SURFACE	gambling	20	0.44
SURFACE	gambling	40	0.88
SURFACE	relational	20	0.22
SURFACE	relational	40	0.65
SURFACE	social	20	0.40
SURFACE	social	40	0.97
SURFACE	wm	20	0.98
UKB	emotion	20	0.58
VOL	gambling	20	0.38
VOL	gambling	40	0.90
VOL	relational	20	0.20
VOL	relational	40	0.60
VOL	social	20	0.76
VOL	wm	20	0.96

Table S1: Proportion of Studies with Peaks Above Threshold. Entries with Proportions equal to 1 are excluded.

References

Alfaro-Almagro, F., Jenkinson, M., Bangerter, N. K., Andersson, J. L., Griffanti, L., Douaud, G., Sotiropoulos, S. N., Jbabdi, S., Hernandez-Fernandez, M., Vallee, E., Vidaurre, D., Webster, M., McCarthy, P., Rorden, C., Daducci, A., Alexander, D. C., Zhang, H., Dragonu, I., Matthews, P. M., . . . Smith, S. M. (2018). Image processing and Quality Control for the first 10,000 brain imaging datasets from UK Biobank. *NeuroImage*, *166*, 400–424. https://doi.org/10.1016/j.neuroimage.2017.10.034

Barch, D. M., Burgess, G. C., Harms, M. P., Petersen, S. E., Schlaggar, B. L., Corbetta, M., Glasser, M. F., Curtiss, S., Dixit, S., Feldt, C., Nolan, D., Bryant, E., Hartley, T., Footer, O., Bjork, J. M., Poldrack, R., Smith, S., Johansen-Berg, H., Snyder, A. Z., & Van Essen, D. C. (2013). Function in the human connectome: Task-fMRI and individual differences in behavior. *NeuroImage*, *80*, 169–189. https://doi.org/10.1016/j.neuroimage.2013.05.033

Binder, J. R., Gross, W. L., Allendorfer, J. B., Bonilha, L., Chapin, J., Edwards, J. C., Grabowski, T. J., Langfitt, J. T., Loring, D. W., Lowe, M. J., Koenig, K., Morgan, P. S., Ojemann, J. G., Rorden, C., Szaflarski, J. P., Tivarus, M. E., & Weaver, K. E. (2011). Mapping anterior temporal lobe language areas with fMRI: A multicenter normative study. *NeuroImage*, *54*(2), 1465–1475. https://doi.org/10.1016/j.neuroimage.2010.09.048

Buckner, R. L., Krienen, F. M., Castellanos, A., Diaz, J. C., & Yeo, B. T. T. (2011). The organization of the human cerebellum estimated by intrinsic functional connectivity. *Journal of Neurophysiology*, *106*(5), 2322–2345. https://doi.org/10.1152/jn.00339.2011

Castelli, F., Happé, F., Frith, U., & Frith, C. (2013). Movement and mind: A functional imaging study of perception and interpretation of complex intentional movement patterns. In *Social neuroscience* (pp. 155–169). Psychology Press.

Delgado, M. R., Nystrom, L. E., Fissell, C., Noll, D. C., & Fiez, J. A. (2000). Tracking the Hemodynamic Responses to Reward and Punishment in the Striatum. *Journal of Neurophysiology*, *84*(6), 3072–3077. https://doi.org/10.1152/jn.2000.84.6.3072

Drobyshevsky, A., Baumann, S. B., & Schneider, W. (2006). A rapid fMRI task battery for mapping of visual, motor, cognitive, and emotional function. *NeuroImage*, *31*(2), 732–744. https://doi.org/10.1016/j.neuroimage.2005.12.016

Smith, R., Keramatian, K., & Christoff, K. (2007). Localizing the rostrolateral prefrontal cortex at the individual level. *NeuroImage*, *36*(4), 1387–1396. https://doi.org/10.1016/j.neuroimage.2007.04.032

Spisák, T., Spisák, Z., Zunhammer, M., Bingel, U., Smith, S., Nichols, T., & Kincses, T. (2019). Probabilistic TFCE: A generalized combination of cluster size and voxel intensity to increase statistical power. *NeuroImage*, *185*, 12–26. https://doi.org/10.1016/j.neuroimage.2018.09.078

Thomas Yeo, B. T., Krienen, F. M., Sepulcre, J., Sabuncu, M. R., Lashkari, D., Hollinshead, M., Roffman, J. L., Smoller, J. W., Zöllei, L., Polimeni, J. R., Fischl, B., Liu, H., & Buckner, R. L. (2011). The organization of the human cerebral cortex estimated by intrinsic functional connectivity. *Journal of Neurophysiology*, *106*(3), 1125–1165. https://doi.org/10.1152/jn.00338.2011

Wheatley, T., Milleville, S. C., & Martin, A. (2007). Understanding Animate Agents: Distinct Roles for the Social Network and Mirror System. *Psychological Science*, *18*(6), 469–474. https://doi.org/10.1111/j.1467-9280.2007.01923.x
